# Epidemiology of Hypertension Stages in Two Countries in Sub-Sahara Africa: Factors Associated with Hypertension Stages

**DOI:** 10.1155/2015/959256

**Published:** 2015-10-01

**Authors:** Kirubel Zemedkun Gebreselassie, Mojgan Padyab

**Affiliations:** ^1^Epidemiology and Global Health, Department of Public Health and Clinical Medicine, Umeå University, 901 85 Umeå, Sweden; ^2^Centre for Population Studies, Ageing and Living Conditions Programme, Umeå University, Umeå, Sweden

## Abstract

Studies using the revised hypertension classification are needed to better understand epidemiology of hypertension across full distribution. The sociodemographic, biological, and health behavior characteristics associated with different stages of hypertension in Ghana and South Africa (SA) were studied using global ageing and adult health (SAGE), WAVE 1 dataset. Blood pressure was assessed for a total of 7545 respondents, 2980 from SA and 4565 from Ghana. Hypertension was defined using JNC7 blood pressure classification considering previous diagnosis and treatment. Multivariate multinomial logistic regression analysis using Stata version 12 statistical software was done to identify independent predictors. The weighted prevalence of prehypertension and hypertension in Ghana was 30.7% and 42.4%, respectively, and that of SA was 29.4% and 46%, respectively, showing high burden. After adjusting for the independent variables, only age (OR = 1.32, 95% CI: 1.14–1.53), income (OR = 1.9, 95% CI: 1.04–3.47), and BMI (OR = 1.16, 95% CI: 1.1–1.22) remained independent predictors for stage 1 hypertension in Ghana, while, for SA, age (OR = 2.27, 95% CI: 1.53–3.36), sex (OR = 0.28, 95% CI: 0.08–1), and BMI (OR = 1.15, 95% CI: 1.07–1.25) were found to be independent predictors of stage 1 hypertension. Healthy lifestyle changes and policy measures are needed to promptly address these predictors.

## 1. Introduction

Worldwide prevalence estimates for hypertension may be as much as 1 billion individuals, and approximately 7.1 million deaths per year may be attributable to hypertension. The World Health Organization reports that suboptimal systolic blood pressure (SBP) >115 mmHg is responsible for 62 percent of cerebrovascular disease and 49 percent of ischemic heart disease (IHD), with little variation by sex [1]. Hypertension has been identified as the leading risk factor for developing congestive heart failure [2], stroke [3], chronic kidney disease, and end stage renal disease [4] and is ranked third as a cause of disability-adjusted life-years [5].

The risk of developing these complications depends on the level of elevated blood pressure and has been seen in all age groups starting from blood pressure as low as SBP 115 and DBP of 75 [6]. Data from observational studies involving more than 1 million individuals have also indicated that death from both IHD and stroke increases progressively and linearly from levels as low as 115 mmHg SBP and 75 mmHg DBP upward especially in individuals ranging from 40 to 89 years of age, indicating need for new blood pressure classification [6]. The risk of coronary heart disease increased significantly in the high range prehypertension individuals (SBP 130–139 or DBP 85–89 mmHg) but not in the low range prehypertensive population (SBP from 120 to 129 or DBP 80 to 84 mmHg) [7].

Because of the new data on lifetime risk of hypertension and the highly increased risk of cardiovascular morbidity associated with levels of BP previously considered to be normal, the JNC 7 report has introduced a new classification that includes the term “prehypertension” for those with BPs ranging from 120 to 139 mmHg systolic and/or 80 to 89 mmHg diastolic. This new designation is intended to identify those individuals in whom early intervention by adoption of healthy lifestyles could reduce BP, decrease the rate of progression of BP to hypertensive levels with age, or prevent hypertension entirely [8]. Robust population-based data using these recent blood pressure categories are still needed to confirm prior estimates and inform policy decision makers in Sub-Saharan Africa.

Increasing urbanization has fueled social and economic changes in Sub-Saharan Africa, which have contributed to a surge in noncommunicable disease (NCD), including hypertension [9]. Epidemiological studies on hypertension in this region have been conducted over the years in an attempt to estimate the burden of hypertension, and these have reported variable rates within and between different population groups. In the first national Demographic and Health Survey, of 12,952 randomly selected South Africans aged 15 years, a high risk of hypertension was associated with less than tertiary education, older age groups, overweight and obese people, excess alcohol use, and a family history of stroke and hypertension [10]. Prehypertension was also more common in those aged 35 years compared with those aged <35 years and in overweight and obese people compared with people of normal weight [11]. Hypertension was defined in these studies as individuals with self-reported treated hypertension or with an average of 2 blood pressure measurements of at least 140/90 mmHg [9, 12, 13].

Prior studies on hypertension mainly focused on these dichotomous definitions of hypertension and did not examine the sociodemographic characteristics and risk factors for hypertension across full distribution of blood pressure. The current study follows the work of Basu and Millet on social epidemiology of hypertension in low- and middle-income countries from World Health Organization's Study on global AGEing and adult health (SAGE) [13]. Their work further showed additional variation in hypertension prevalence and social determinants of awareness when categorical definitions of hypertension were used compared to dichotomous definitions. Men had significantly lower probability of hypertension awareness than women at stage 1, but not at stage 2 [14]. This empirical study with an objective of studying social determinants and risk factors of different stages of hypertension tries to address this question. It determines the prevalence and independent predictors of different stages of hypertension in two countries in Sub-Saharan Africa, Ghana and South Africa.

## 2. Methods

### 2.1. Study Design and Population

We conducted a cross-sectional analysis of the Study on global AGEing and adult health (SAGE), WAVE 1 dataset which is a part of longitudinal survey program in WHO's multicountries study unit. The main SAGE surveys compile comparable longitudinal information on the health and well-being of adult populations and the ageing process from nationally representative samples in six middle- and low-income countries. We used the dataset of two Sub-Saharan countries, Ghana and South Africa. The SAGE study included nationally representative sample of persons aged 50 years and sample from younger adults aged 18–49 years as comparison which were also selected from both countries. Multistage cluster sampling was used [15]. The sampling design entailed two-stage probabilistic sample that yielded national and subnational estimates. In the first stage, a total sample of 600 enumeration areas (EA) in South Africa and 300 (EA) in Ghana were drawn from the master sample and used as the primary selection units (PSU). The second stage of the sample design was the selection of households from the EA's which formed the secondary sampling units. This stage of the process involved georeferenced aerial photograph maps of urbanized areas on which the locations of households were plotted. The total sample size of individuals was targeted to be 1000 people in the age group 18–49 years and 5000 people aged 50 years or older in each country.

### 2.2. Questionnaire

We recorded information about the household members from household and individual questionnaires; living place variable from the household questionnaire and sociodemographic characteristics, work history, blood pressure measurements, and risk factors including preventive health behaviors were taken from the individual questionnaire. Both the household and individual questionnaires were translated into six local South African and three local languages of Ghana. The respondents were interviewed face to face.

### 2.3. Blood Pressure Measurements and Classification

Blood pressure was measured three times while the respondent seated using automated OMRON R6 Wrist Blood Pressure Monitor, HEM-6000-E, Health Care Europe, B.V., Hoofddorp, The Netherlands. Such digital monitors have been shown to have high degree of agreement with mercury sphygmomanometers for systolic blood pressure [16, 17]. Average blood pressure was calculated arithmetically for the 3 measurements of each systolic and diastolic blood pressure. Missing values were excluded from being included in the study. Blood pressure classification was done using JNC 7 algorithm [1]. Prehypertension was defined as systolic blood pressure (SBP) measurement of 120–139 mmHg or diastolic blood pressure (DBP) of 80–89 mmHg. Stage 1 hypertension was defined as SBP of 140–159 mmHg or DBP of 90–99 mmHg and stage 2 as SBP of greater than or equal to 160 mmHg or DBP of greater than or equal to 100 mmHg. Accordingly normal pressure was defined as SBP of less than 120 mmHg and DBP of less than 80 mmHg.

All respondents were initially asked if they have ever been diagnosed with hypertension and if they did, whether or not they have been taking any kind of drugs or other treatment for the last 2 weeks and last 12 months. Normal blood pressure was defined as SBP <120 and DBP <80 mmHg and either no previous history of diagnosis or not taking antihypertensive medication. Those with SBP of 120 or more and DBP of 80 or more who did not have prior diagnosis nor treatment were designed as to have hypertension. They were subsequently stratified into three mutually exclusive different stages of hypertension. Those who reported as either having a diagnosis of hypertension previously or taking antihypertensive medication were excluded from analysis due to overlapping.

### 2.4. Sociodemographic Characteristics

All participants older than 18 years, of both sexes, were included in the study and classified as living in urban or rural area. Current marital status (married or single that included unmarried, widowed, or separated) was asked about using the individual questionnaire. Educational level was assessed by first asking whether respondents have been in any school, and those who answered yes were asked for the highest educational level completed according to the international standard classification of education [18]. Respondents were asked whether they had ever worked for pay, type of work, and employer. Occupation responses were written verbatim by the interviewer, then coded, and mapped to the International Standard Classifications of Occupations (ISCO) scheme [19]. Classification of income quintiles was based on permanent income estimates derived from household assets and characteristics of the dwelling upon which The 2001 WHO World Health Survey/SAGE WAVE 0 relied [15]. Recent alcohol intake was asked for by asking whether the respondents have consumed alcohol in the past 30 days. Response was documented as yes and no. Current smoking (yes or no), with provision for the collection of information on other forms of smoking apart from cigarettes, such as cigars, pipes, snuff, or chewing smokeless tobacco, was asked about. Question was formulated based on guidelines for controlling and monitoring the tobacco epidemic [20]. Work related and sport or leisure time physical activities were separately asked in a typical week. Vigorous or moderate physical activities required hard or moderate physical effort and caused an increase in breathing or heart rate for at least 10 minutes [21]. Fruit and vegetable servings in typical 24 hours were asked about. Inadequate intake was defined based on WHO recommendations and labeled as less than 5 servings (80 g per serving) on a typical day [22].

### 2.5. Anthropometric Measurements

Height was measured in centimeters after the respondents took off their shoes, put their feet and heels close together, stood straight, and stood forward with their back, head, and heels touching the wall. Next body weight was measured in kilograms. Body composition and fatness were assessed using WHO body mass index (BMI) derived from measured weight in kilograms and normalized by dividing by height in meters squared. It was categorized as underweight <18.5, normal weight 18.5–24.9, overweight 25 to 29.9, and obese greater than or equal to 30. Since abdominal obesity is highly correlated with atherosclerotic cardiovascular morbidity and mortality than BMI indices [23], waist circumference was also used to measure central obesity. The interviewer identified the top of the hip bone and after making sure the tape measure is parallel to the floor all the way around the body measured waist circumference (WC). The National Cholesterol and Education Program: Adult Treatment Panel III (NCEP: ATP III) guideline was used to designate central obesity: accordingly men with WC measurements greater than or equal to 102 cm and women with greater than or equal to 88 cm were considered to have one [24].

### 2.6. Statistical Analysis

Stata version 12 statistical software was used to analyze data after being cleaned. The individual and household data were merged together. Living place variable was taken from the household data while all the others were included from the individual dataset. To make sure the results of the individual country dataset represent the respective country population, weighting at the country level was done which was available in the SAGE dataset. Individual weights were poststratified according to the 2009 projected population estimates in Ghana and to the 2009 medium midyear population estimates in South Africa [25]. Weighted estimates of different stages of hypertension prevalence were reported as proportions of the actual sample size. Invalid blood pressure measurements such as values of diastolic blood pressure greater than systolic and those in the outliers were considered missing and excluded from analysis.

The associations between sociodemographic, biological, and health behavior variables and stages of hypertension (prehypertension, stage 1, and stage 2) were assessed in a two-step procedure where individuals with the different stages of hypertension were compared separately with those having normal blood pressure. In the first step, each variable was evaluated independently in a bivariate multinomial logistic regression analysis with different stages of hypertension as dependent variable to generate unadjusted OR with respondents' characteristics in each country separately. Those variables with *P* values less than 0.2 were retained and entered into multinomial logistic regression model in ascending stepwise manner to determine variables that were independently associated with the stages of hypertension. A probability level of *P* < 0.05 was considered significant. Age and BMI variables were entered in the multivariable multinomial models as continuous due to fewer numbers of people in the most upper and lower categories. All other variables were retained as categorical.

### 2.7. Ethical Approval

Informed consent was obtained from each respondent for interviews and measurements of anthropometrics. SAGE study received ethical clearance from WHO ethical review committee [26].

## 3. Results

The total sample size of the study was 8939, 3974 from South Africa and 4965 from Ghana. Participants who have been diagnosed previously with high blood pressure or who were already taking treatment were excluded from analysis due to overlap and difficulty to stratify them into mutually exclusive hypertension stages. Those with previous diagnosis of hypertension were 587 (12%) in Ghana and 1,111 (28%) in South Africa. Individuals taking antihypertensive medications were 396 (8%) in Ghana and 981 (25%) in South Africa. Completed interview response in South Africa was 2853 (96%) and 5057 (99%) in Ghana. Of these blood pressure was assessed for a total of 7545, 2980 (75%) respondents from South Africa and 4565 (92%) from Ghana which was included for analysis (Table 1). A higher number of study participants in Ghana were males (50.2%) compared to 48.5% in South Africa. More people lived in Urban areas in South Africa (69.7%) compared to only 44.4% in Ghana. In South Africa, the proportion of obesity was 30.5% as compared to 12.1% in Ghana. Income quintile distribution was similar in both countries. Only 26.1% of the respondents in Ghana and 24.1% in South Africa fulfilled the definition of normal blood pressure. Prehypertension was more prevalent in Ghana (30.7%) than South Africa (29.4%). The weighted prevalence of hypertension (both stages 1 and 2) was 42.4% in Ghana and 46% in South Africa. The age group distribution across stages of hypertension was similar in both countries, the majority of prehypertensive individuals being in the age group of 18 to 49. Obese individuals constituted 45.4% of stage 2 hypertension in South Africa while they constituted only 25.3% of stage 2 hypertension in Ghana.

Prehypertension was significantly associated in Ghana with income distribution (OR = 1.9, 95% CI: 1.11–3.23) and BMI category (OR = 2.64, 95% CI: 1.11–6.3) in the bivariate multinomial analysis when compared with normal blood pressure measurement while in South Africa age (OR = 2.66, 95% CI: 1.34–5.28) and educational level (OR = 0.18, 95% CI: 0.05–0.63) only had a significant association (Table 2). Income and BMI remained to have a significant association with stage 1 hypertension. In addition age emerged as a new variable with significant correlation with stage 1 hypertension in Ghana in the bivariate analysis (OR = 1.71, 95% CI: 1.25–2.33). In South Africa stage 1 hypertension was significantly associated with age of the study participants (OR = 6.02, 95% CI: 2.61–13.88), educational level (OR = 0.09, 95% CI: 0.02–0.49), type of occupation (OR = 4.32, 95% CI: 1.06–17.55), BMI category (OR = 11.72, 95% CI: 2.35–58.43), and number of vegetable servings per day in the bivariate multinomial analysis (OR = 0.3, 95% CI: 0.11–0.82). Age, income, and BMI remained to be significantly associated with stage 2 hypertension in the bivariate multinomial analysis in Ghana. In addition educational level (OR = 0.07, 95% CI: 0.02–0.35) became a significant correlate with stage 2 hypertension. In South Africa factors significantly associated with stage 2 hypertension were age, living place, educational level, and fruit and vegetable intake.

## 4. Multinomial Logistic Regression Model with Pooled Multivariable Analysis

### 4.1. Ghana

**Figure 1:**
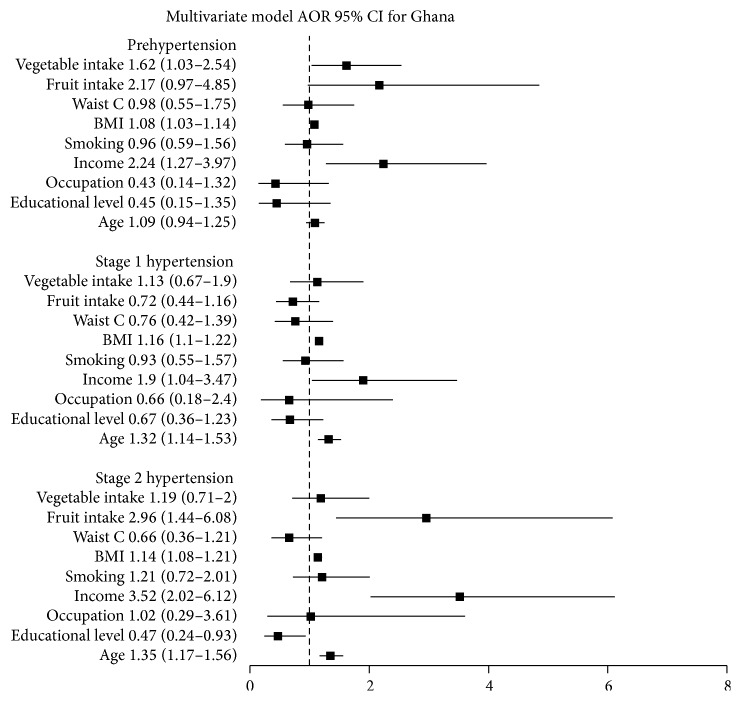
Adjusted odds ratios and 95% confidence intervals for stages of hypertension, Ghana.

For the multinomial logistic regression analysis, 10 independent variables which were associated with hypertension stages at level of *P* value < 0.2 in the bivariate analysis were retained in the model. These were age, educational level, type of occupation, income quintile, smoking status, BMI category, abdominal waist circumference, work related physical activity, and fruit and vegetable intake per day. Those with *P* values <0.05 in the multivariable model were considered statistically significant and were considered independent predictors of hypertension stages (Figure 1). At prehypertension level BMI (OR = 1.08, 95% CI: 1.03–1.14), income (OR = 2,24, 95% CI: 1.27–3.97), and number of vegetable intakes per day were found to be independent predictors after being adjusted for the other variables in the model. Income and BMI remained independent predictors for stage 1 hypertension as well. There was 32% increased risk of having stage 1 hypertension for every age increase by one year (OR = 1.32, 95% CI: 1.14–1.53). Age (OR = 1.35, 95% CI: 1.17–1.56), income (OR = 3.52, 95% CI: 2.02–6.12), and BMI (OR = 1.14, 95% CI: 1.08–1.21) remained as independent predictors for stage 2 hypertension as well. In addition higher educational level emerged as a protective variable against stage 2 hypertension in the model (OR = 0.47, 95% CI: 0.24–0.93).

### 4.2. South Africa

**Figure 2:**
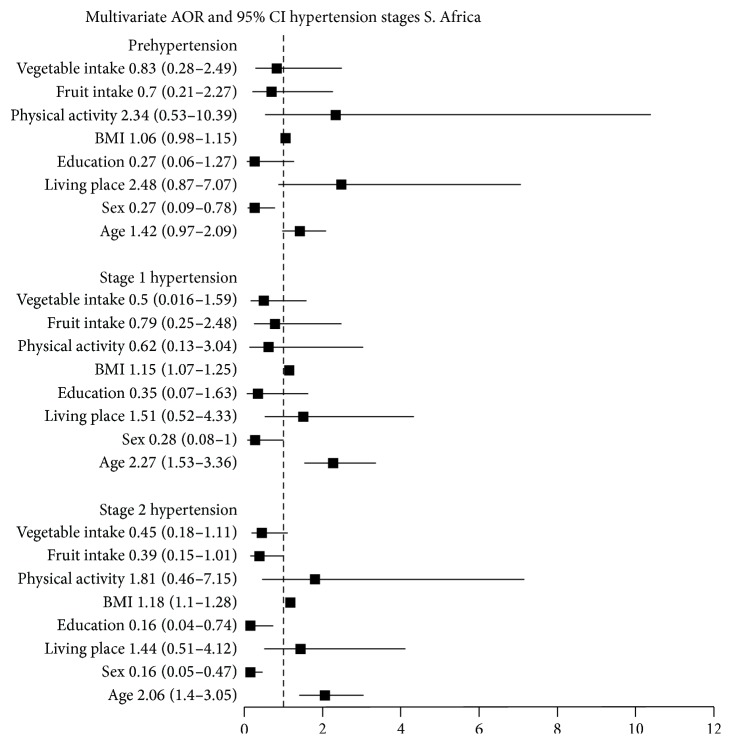
Adjusted odds ratios and 95% confidence intervals for stages of hypertension, South Africa.

Eight variables which were associated with stages of hypertension in the bivariate analysis at probability level of *P* < 0.2 were retained in the multinomial logistic regression model. These were age, sex, living place, educational level, BMI category, work related physical activity, and fruit and vegetable servings per day (Figure 2). Only sex (OR = 0.27, 95% CI: 0.09–0.78) remained independent predictor of prehypertension in the multivariate logistic regression model after being adjusted for the other variables. Age and BMI emerged as independent predictors of stage 1 hypertension (OR = 2.27, 95% CI: 1.53–3.36 and OR = 1.15, 95% CI: 1.07–1.25). Sex remained as an independent predictor of stage 1 hypertension (OR = 0.28, 95% CI: 0.08–1). Being female resulted in 72% lower risk of having stage 1 hypertension than being male (OR = 0.28, 95% CI: 0.08–1). Age (OR = 2.06, 95% CI: 1.4–8.7), sex (OR = 0.16, 95% CI: 0.05–0.47), and BMI (OR = 1.18, 95% CI: 1.1–1.28) remained independent predictors of stage 2 hypertension in the final model. Educational level emerged as an independent predictor of stage 2 hypertension. Those with high school education tended to have 84% lower odds of stage 2 hypertension compared to those with no education (OR = 0.16, 95% CI: 0.04–0.74).

## 5. Discussion

The study has showed high burden of prehypertension and hypertension stages in the Sub-Saharan African countries, Ghana and South Africa. The weighted prevalence of hypertension (including both stages 1 and 2) was higher in South Africa (46%) compared to Ghana (42.4%). The high prevalence of hypertension in the current study is mostly due to higher age distribution of participants. More than 85% of respondents in Ghana and around 90% South Africans were in the age group more than 50, as part of the WHO SAGE study. The probability that a middle-aged or elderly individual will develop hypertension in his or her lifetime is 90% [27], explaining the higher prevalence in this study. Higher prevalence of hypertension (77.3%) than the current study was also reported in hypertension and associated factors in older adults study in South Africa [28]. Comparing the two countries South Africa had higher prevalence of hypertension that could be explained by higher proportion of people living in urban area (69.7% in South Africa and 44.4% in Ghana) and increased number of obese people (30.5% in South Africa and 12.1% Ghana). In addition lower rate of physical activity and fruit intake per day was reported in South Africa.

The weighted prevalence of prehypertension was higher in Ghana (30.7%) than South Africa (29.4%). Higher reports were made from the PURE hypertension trial done on 153,996 adults aged 35 to 70 years, from 628 communities in 3 high-income, 10 upper-middle and low-middle-income, and 4 low-income countries of 36.8% [12]. These are groups of people with increased cardiovascular risk but who do not need pharmacologic treatment unless there is another compelling medical indication such as diabetes or chronic kidney disease [29]. They have higher likelihood of progression to overt hypertension and need lifestyle changes as a treatment such as weight reduction, physical activity, and decreased salt intake [30]. Progression from prehypertension to stage 1 hypertension was positively related to male gender, higher waist circumference, and having parents with hypertension in population-based study Keelung, Taiwan [31]. Identifying these individuals has high public health importance as such measures taken could delay or prevent progression or development of hypertension.

The weighted prevalence of stage 1 hypertension in Ghana was higher (21.6%) than in South Africa (18.5%) and the weighted estimate of stage 2 hypertension was higher in South Africa (28.1%) and Ghana (21.6%). Prior studies on these two countries were mainly done on dichotomized blood pressure classification using SBP of 140 mmHg or DBP of 90 mmHg and more. According to hypertension in South African adults, results from the Demographic and Health Survey, 1998, the prevalence rate of hypertension was 11% in males and 14% in females when blood pressure cut-off point (160/95 mmHg) was used [32]. In the current study BMI was an independent predictor for prehypertension in both Ghana and South Africa which was a similar finding in the study done in the Ashanti region of Ghana which showed age (OR = 1.56, 95% CI: 1.12–2.18; *P* < 0.01), obesity (OR = 2.71; 95% CI: 1.40–5.24; *P* < 0.001), and sex (OR = 2.36, 95% CI: 1.77–3.15; *P* < 0.001) being independent predictors of prehypertension on multivariable logistic regression [11]. In addition income became one of the strongest predictors for prehypertension in Ghana in our study, with higher income quintiles associated with higher levels of prehypertension. This was similar with household characteristics for older adults and study background from SAGE Ghana WAVE 1, which showed people with the higher income quintiles generally reported more hypertension (income quintile 5, wealthiest = 26.7%, versus quintile 1, poorest = 5.5%) and received more current and chronic therapy [15]. Other studies in US and Canada have shown the opposite effect; inverse linear relationship between household income and hypertension prevalence in the United States, but no evidence of such a relationship in Canada, was seen due to similar burden of hypertension across different socioeconomic classes [33]. Middle-income was a high correlate in another study in urban India [34]. Getting income data reliably was difficult as income was generated from household assets and converted later to quintiles, hence requiring cautious interpretation of result. The proportion of both overweight and obese was higher in the 4th and 5th income quintiles than the lower ones (23% and 30%, resp.) which could explain the high prevalence of prehypertension in the group.

Being in the higher BMI category contributed significantly to having prehypertension and could be related to the low physical activity of the current study participants [35]. Those with the lowest work related physical activity had the highest rates of prehypertension. South Africa has one of the lowest rates of insufficient physical activity (49% in adult women and 43% in adult men) compared to global figure of 17% and Africa's coverage of about 10% [36]. Some of the explanations were high rate of urbanization, increased mechanized labour, and television watching [37]. The very low report of physical activity contributing to both obesity and prehypertension needs to be addressed in South Africa by clinicians, public health specialists, patients, and policy makers at the government level.

We have tried to see the social determinant and risk factors for different hypertension stages (1 and 2) separately and whether they differ in the two countries and across different hypertension stages in the same country. The study has showed only age, income, and BMI remaining as independent predictors of stage 1 hypertension in Ghana. We found educational level and number of fruit intakes per day having an inverse relationship to stage 1 hypertension in South Africa in the bivariate multinomial analysis. This is similar with the first demographic and health survey study: determinants and treatment of hypertension in South Africa which showed higher risk of having hypertension with less than tertiary education [10]. Adults with no education or less than primary school were more than 50%, and the highest report of stage 1 hypertension was seen in this group in the current study. Curriculum reforms and models to increase opportunity of education in the postapartheid education system are undergoing [38] and should be further strengthened.

Increasing fruit and vegetable intake was seen to have significant blood pressure lowering effect in stage 1 and stage 2 hypertension. This effect was not seen in the final model. The finding is consistent with other trials that demonstrated the greatest benefit of dietary changes at lower stages of hypertension [30]. The DASH (Dietary Approaches to Stop Hypertension) trials introduced the DASH diet which is comprised of four-five servings of fruit, four-five servings of vegetables, two-three servings of low-fat dairy per day, and <25 percent fat [39]. Drop in average systolic blood pressure by 11.4 mmHg and diastolic blood pressure by 5.5 mmHg in hypertensive individuals has been seen as early as two weeks, with the DASH diet. Increasing fruit intake is thus highly recommended in individuals especially with lower stages of hypertension to prevent progression of disease. Countries should be working on ways to make availability of fruits accessible and affordable. The weighted estimate of people taking the recommended fruit intake was very low (9.9%) in Ghana and (3.5%) South Africa. Vegetable intake was even much worse (0.9%) in Ghana and (3.5%) in South Africa. The wide confidence interval and attenuated beneficial effects of fruits and vegetables could be attributed to the very little proportion of people in these categories making the expected statistical association loose.

Though smoking cigarette was one of the most important risk factors for cardiovascular disorders and acute myocardial infarction in the INTERHEART Africa study [40], it was associated only in the bivariate analysis when studied individually. The effect was lost when multiple variables were retained in the regression model in both countries at every stage of hypertension. The incidence of hypertension increases in those who smoke 15 or more cigarettes per day [41] and could be the reason why strong association was not seen as only less than 2% individuals (Ghana) and around 7% (South Africa) smoked more than 15 cigarettes per day. Other studies have also documented “lower blood pressure measurements in those habitual smokers than nonsmokers due to weight loss and some vasodilatory effect of cotinine a metabolite of nicotine” [42, 43].

Only increasing age, income, BMI, and educational level were found to be independent risk factors for stage 2 hypertension in the final model in Ghana. There was no significant variation of stage 2 hypertension between sexes in Ghana, although it is known that men had higher systolic blood pressure measurements in early adulthood, while older women have steeper age-related rate of rise [27]. This could be in part due to the higher proportion of women who are overweight and obese in both countries.

This particular study has assessed the burden of hypertension in Sub-Saharan African countries. It focused mainly on risk factors and social determinants across different stages of hypertension among the two countries. This helps to identify newly emerging associated risk factors at different stages of hypertension and helps to be able to recommend measures accordingly. As it is always said “Prevention is better than Cure,” we also recommend identifying individuals and treating accordingly at pre- and early hypertension stages, where the maximum benefit of lifestyle changes can be seen. These include having regular and intense physical exercise, reduction of body weight which can decrease average SBP by 5–20 mmHg for every 10 kg weight loss [1], increasing the opportunity to have basic education for all, and educating people with higher income in Ghana who are at high risk of prehypertension and subsequent overt hypertension due to probable acculturation to change their lifestyle. Government bodies at policy level and health specialists need to design methods to improve diagnosis and treatment of hypertension at earlier stages in respective countries.

## 6. Limitation

One of the major limitations of the current study was high number of missing and some invalid values in the original dataset. Missing and invalid blood pressure measurements accounted for 8% in Ghana and 25% in South Africa. They were excluded during the data cleaning period and not included in analysis. Alcohol consumption was an independent variable with the highest missing values (63%) in Ghana and (35%) in South Africa. This could be the reason for not seeing the expected protection from moderate dose of alcohol against hypertension and increased risk from excess dose [44]. The other components of therapeutic lifestyle changes that include intake of salt, saturated fat, and amount of calories [45] were not included in the SAGE questionnaire and their association with hypertension was not studied. The current study as any other cross-sectional studies determines only associations that are statistically significant without inferring causality. Further cohort studies that examine risk and causality are recommended. And finally using the JNC 7 blood pressure classification that was originally designed for US population and using its blood pressure cut-off points for Sub-Saharan population could impose risk of overgeneralization. Blood pressure cut-off points at which cardiovascular morbidity starts should be looked for in Sub-Saharan Africa context and guidelines should develop in the future.

## Figures and Tables

**Table 1:** Sociodemographic, health behavior, and biological characteristics (%) of study subjects, by country and hypertension stages.

*N* = 7545	Ghana (*n* = 4565)					South Africa (*n* = 2980)				
	Normal	Pre-HTN	Stage 1 HTN	Stage 2 HTN	Total (%)	Normal	Pre-HTN	Stage 1 HTN	Stage 2 HTN	Total (%)
**Sociodemographic variables**										
Age groups (years)	∗∗					∗∗				
18–49	28.1	32.6	20.5	18.8	762 (76.9)	28.2	31.7	16.2	23.9	334 (79.7)
50–59	20.1	23.2	28.3	28.4	1532 (9.4)	9.1	20.9	24.9	45.1	1242 (11.1)
60–69	18.5	24.7	23.1	33.7	1055 (6.3)	6.9	20.6	28.5	44.1	803 (5.7)
70–74	18.4	26.7	23.9	30.9	837 (5.1)	9.5	15	33.1	42.4	424 (2.5)
80+	19.0	27.6	24.5	29	374 (2.3)	5.8	20.4	29.6	44.2	171 (1.1)
Sex						*∗*				
Male	26.2	29.3	20.7	23.8	2466 (50.2)	16.4	33.9	18.5	31.2	1361 (48.5)
Female	25.9	32.1	22.6	19.4	2099 (49.8)	31.3	25.1	18.4	25.3	1617 (51.5)
Living place						*∗*				
Urban	23.6	30.3	24	22.2	1747 (44.4)	28.3	28.6	18.3	24.9	1890 (69.7)
Rural	28	31.1	19.8	21.1	2818 (55.6)	14.7	31.4	18.2	35.7	1084 (30.3)
Marital status										
Married	25.6	28.1	23.8	22.5	1758 (26.6)	28.9	29.1	19.5	22.6	1346 (46.5)
Single	26.3	31.8	20.8	21.1	2782 (73.4)	20	29.4	17.8	32.8	1578 (53.6)
Educational level	*∗*					∗∗				
No school	21.9	27.6	22.1	28.4	2337 (31.1)	7.7	20.8	22.4	49.1	647 (7.3)
<6 yrs	29.3	30.3	23.5	17	498 (12.8)	5.5	23.4	22.2	48.9	556 (13.9)
Primary	26.4	29.3	24.8	19.6	567 (20.1)	10.4	28.9	22	38.7	557 (13.9)
Secondary	21.8	41.3	21	15.8	244 (10.9)	35.2	26.2	17.4	21.3	391 (27.7)
High school	29.5	32.3	18.2	20.1	737 (20.7)	40.3	19.2	18.4	22.1	221 (29.1)
University	36.8	28.8	18.2	16.3	154 (4.4)	41.7	27.1	11.6	19.6	143 (8.1)
Occupation	*∗*									
Public sector	23.6	34.1	21.8	20.6	380 (7.2)	26.9	30.1	10.7	32.4	395 (19.8)
Private	26.7	20.1	24.1	29.1	180 (5.2)	13.4	32.7	23	31	1421 (53.1
Self-employed	25	32.3	22	20.7	3564 (80.8)	44	31.8	10.5	13.8	106 (4)
Informal	25.2	24.0	15	35.8	332 (6.8)	33.9	16.9	18.9	30.3	592 (23.1)
Income	∗∗									
Quintile 1 (poorest)	33.4	28.3	21.5	16.9	939 (15.6)	20	30	21.2	28.8	640 (19.6)
Quintile 2	33	29	16.9	21.1	932 (18)	28.3	23.6	22.8	25.3	631 (20)
Quintile 3	19.3	31	20.8	28.8	922 (19.4)	27.4	19.6	17.3	35.6	545 (20.2)
Quintile 4	18.8	34.8	24.6	21.8	921 (22.2)	21.1	35.6	13	30.4	551 (19.5)
Quintile 5 (richest)	28.2	29.6	23.2	19.1	845 (24.9)	23.7	37.7	17.7	20.9	595 (20.8)
**Biological variables**										
BMI (kg/m^2^)	∗∗					∗∗				
Underweight	31	33	14.4	21.5	670 (9.3)	21.1	35.7	8.2	35	130 (3.5)
Normal	30.6	29.4	19.8	20.2	2543 (54.4)	35.9	35.6	12.2	16.4	777 (36.1)
Overweight	21.4	32.8	22.2	23.6	889 (24.2)	32.4	21.2	21.4	25	849 (30)
Obese	10.8	30.4	33.4	25.3	444 (12.1)	5.7	23.2	25.7	45.4	1148 (30.5)
Waist circumference	*∗*									
Normal	27.9	29.9	20.2	22	3608 (76.7)	24.5	32	18.4	25.1	1574 (63.2)
Abnormal	20.2	32.8	26.2	20.9	930 (23.3)	20	25.2	20.8	34	1180 (36.9)
**Health related behaviors**										
Smoking	*∗*									
No	25.9	31.4	22.2	20.5	3416 (84.1)	26.5	32.3	16.9	24.4	1819 (67.6)
Yes	26.2	27.6	18.8	27.5	1143 (15.9)	19.1	23.2	22.2	35.6	1077 (32.4)
Current alcohol use										
No	25.9	31.7	20.7	21.7	1181 (44.3)	25.4	21.2	31.2	22.2	347 (33.5)
Yes	28.2	27.2	22.7	22	1492 (55.7)	14.9	27.8	18.5	38.8	489 (66.5)
Work related physical activity	*∗*					*∗*				
High	25.5	33.5	19.9	21.2	1868 (44.7)	30.7	15.6	27	26.7	306 (12.4)
Moderate	29	28.7	22.5	19.7	1400 (27.5)	33.3	28.1	14.5	24.1	852 (36.3)
Low	23.5	28.5	23.7	24.3	1289 (27.8)	16	33.6	19.3	31.1	1734 (51.4)
Fruit intake/day	*∗*					∗∗				
0-1	25.7	27.9	23.9	22.5	1726 (32.3)	17.6	24.9	21.1	36.4	1476 (46.3)
2–4	28	31.9	19.8	20.3	2374 (57.8)	29.8	34.2	17.4	18.6	1305 (50.2)
>=5	13.7	32.7	24.7	29	402 (9.9)	37.8	9.2	7	46	79 (3.5)
Vegetable intake/day	*∗*					∗∗				
0-1	29.9	24.1	24.5	21.5	1112 (24.9)	14.9	24.8	24.4	35.9	1160 (35.7)
2–4	24.1	32.4	21.2	22.3	3269 (74.3)	30.8	32.7	15.3	21.2	1592 (60.6)
>=5	21.8	36.2	29.8	12.2	57 (0.9)	5.4	10.1	21.7	62.9	116 (3.7)
Blood pressure number	957	1205	1108	1295	4565	301	647	738	1294	2980
Weighted prevalence (%)	26.1	30.7	21.6	21.6		24.1	29.4	18.5	28.1	

^*∗*^ *P* < 0.2, ^*∗∗*^ *P* < 0.05.

*n* = number of observations in the sample.

All percentages (%) are put as weighted estimates of the sample to represent population.

**Table 2:** Association between sociodemographic, health behavior, and biological variables with stages of hypertension (pre-HTN, stage 1, and stage 2) compared to normal blood pressure: bivariate multinomial analysis.

*N* = 7545	Ghana (*n* = 4565)			South Africa (*n* = 2980)		
	Pre-HTN	Stage 1	Stage 2	Pre-HTN	Stage 1	Stage 2
	OR (95% CI)	OR (95% CI)	OR (95% CI)	OR (95% CI)	OR (95% CI)	OR (95% CI)
Age groups (years)						
18–49	1	1	1	1	1	1
50–59	1 (0.78–1.29)	1.93 (1.47–2.54)^*∗*^	2.12 (1.59–2.83)	2.06 (1.04–4.09)	4.77 (2.13–10.67)	5.88 (3.01–11.48)
60–69	1.15 (0.86–1.54)	1.71 (1.25–2.33)^*∗*^	2.72 (2.02–3.67)	2.66 (1.34–5.28)	7.19 (3.38–15.28)	7.57 (3.87–14.82)
70–74	1.25 (0.94–1.66)	1.78 (1.26–2.51)^*∗*^	2.51 (1.8–3.51)	1.4 (0.57–3.44)	6.02 (2.61–13.88^*∗*^)	5.25 (2.39–11.5)^*∗*^
80+	1.25 (0.8–1.97)	1.77 (1.1–2.84)^*∗*^	2.29 (1.47–3.57)^*∗*^	3.17 (1.19–8.43)^*∗*^	8.95 (3.5–22.89)	9.09 (3.77–21.93)
Sex						
Male	1	1	1	1	1	1
Female	1.11 (0.78–0.58)	1.1 (0.75–1.61)	0.82 (0.56–1.21)	0.39 (0.14–1.09)	0.52 (0.19–0.44)	0.43 (0.17–1.06)
Living place						
Urban	1	1	1	1	1	1
Rural	0.86 (0.6–1.24)	0.69 (0.47–1.03)	0.8 (0.51–1.25)	2.12 (0.82–5.49)	1.92 (0.75–4.88)	2.76 (1.14–6.68)^*∗*^
Marital status						
Married	1	1	1	1	1	1
Single	1.1 (0.71–1.72)	0.85 (0.55–1.3)	0.91 (0.59–1.4)	1.46 (0.49–4.34)	1.32 (0.46–3.77)	2.1 (0.8–5.52)
Educational level						
No school	1	1	1	1	1	1
<6 yrs	0.82 (0.45–1.49)	0.8 (0.44–1.44)	0.45 (0.25–0.8)	1.58 (0.38–6.53)	1.39 (0.44–4.34)	1.4 (0.47–4.15)
Primary	0.88 (0.52–1.49)	0.93 (0.53–1.64)	0.57 (0.33–0.98)^*∗*^	1.03 (0.28–3.84)	0.73 (0.2–2.66)	0.58 (0.17–1.95)
Secondary	1.5 (0.77–2.9)	0.95 (0.48–1.92)	0.56 (0.26–1.2)	0.27 (0.07–1.05)	0.17 (0.05–0.61)	0.09 (0.03–0.3)
High school	0.87 (0.52–1.45)	0.61 (0.35–1.07)	0.53 (0.3–0.92)	0.18 (0.05–0.63)^*∗*^	0.16 (0.04–0.7)	0.09 (0.02–0.33)
University	0.62 (0.25–1.56)	0.49 (0.18–1.34)	0.34 (0.11–1.06)	0.24 (0.05–1.13)	0.09 (0.02–0.49)^*∗*^	0.07 (0.02–0.35)^*∗*^
Occupation						
Public sector	1	1	1	1	1	1
Private	0.52 (0.17–1.57)	0.97 (0.29–3.23)	1.25 (0.38–4.09)	2.18 (0.4–12.03)	4.32 (1.06–17.55)^*∗*^	1.93 (0.49–7.64)
Self-employed	0.89 (0.47–1.72)	0.95 (0.49–1.85)	0.95 (0.46–1.97)	0.65 (0.06–7.18)	0.6 (0.07–5.23))	0.26 (0.03–2.33)
Informal	0.66 (0.27–1.62)	0.64 (0.27–1.55)	1.63 (0.6–4.39)	0.45 (0.07–2.99)	1.4 (0.22–8.72)	0.74 (0.16–3.51)
Income						
Quintile 1 (poorest)	1	1	1	1	1	1
Quintile 2	1.04 (0.6–1.79)	0.79 (0.44–1.41)	1.26 (0.74–2.14)	0.56 (0.14–2.26)	0.76 (0.17–3.43)	0.62 (0.18–2.14)
Quintile 3	1.9 (1.11–3.23)^*∗*^	1.67 (0.94–2.96)	2.95 (1.76–4.95)	0.48 (0.1–2.37)	0.6 (0.11–3.15)	0.9 (0.2–4.12)
Quintile 4	2.19 (1.3–3.68)	2.03 (1.14–3.64)^*∗*^	2.29 (1.32–3.97)^*∗*^	1.13 (0.28–4.6)	0.58 (0.15–2.29)	1 (0.27–3.73)
Quintile 5 (richest)	1.24 (0.72–2.12)	1.27 (0.74–2.21)	1.33 (0.73–2.42)	1.06 (0.25–4.43)	0.7 (0.2–2.42)	0.61 (0.18–2.06)
**Biological variables**						
BMI (kg/m^2^)						
Underweight	1	1	1	1	1	1
Normal	0.9 (0.51–1.6)	1.39 (0.76–2.55)	0.95 (0.53–1.71)	0.59 (0.12–2.87)	0.88 (0.16–4.68)	0.27 (0.05–1.38)
Overweight	1.44 (0.76–2.74)	2.23 (1.09–4.56)^*∗*^	1.58 (0.77–3.26)	0.39 (0.07–2.06)	1.71 (0.28–10.53)	0.47 (0.08–2.77)
Obese	2.64 (1.11–6.3)^*∗*^	6.62 (2.79–15.69)	3.37 (1.37–8.26)^*∗*^	2.42 (0.53–11.03)	11.72 (2.35–58.43)^*∗*^	4.81 (0.98–23.54)
Waist circumference						
Normal	1	1	1	1	1	1
Abnormal	1.52 (0.97–2.38)	1.79 (1.12–2.87)^*∗*^	1.31 (0.8–2.13)	0.96 (0.25–3.75)	1.38 (0.38–5.04)	1.66 (0.49–5.56)
**Health related behaviors**						
Smoking						
No	1	1	1	1	1	1
Yes	0.87 (0.56–1.34)	0.83 (0.51–1.36)	1.33 (0.85–2.07)	1 (0.33–3.04)	1.83 (0.57–5.84)	2.02 (0.69–5.95)
Current alcohol use						
No	1	1	1	1	1	1
Yes	1.27 (0.77–2.1)	0.99 (0.6–1.65)	1.08 (0.66–1.76)	0.44 (0.1–1.93)	0.98 (0.15–6.56)	0.33 (0.08–1.42)
Work related physical activity						
High	1	1	1	1	1	1
Moderate	0.75 (0.49–1.15)	1 (0.63–1.58)	0.82 (0.5–1.33)	1.66 (0.35–7.95)	0.5 (0.08–2.93)	0.83 (0.19–3.62)
Low	0.92 (0.57–1.5)	1.29 (0.75–2.23)	1.24 (0.72–2.16)	4.14 (0.9–19.13)	1.38 (0.26–7.42)	2.24 (0.56–8.98)
Fruit intake/day						
0-1	1	1	1	1	1	1
2–4	1.05 (0.72–1.53)	0.76 (0.5–1.16)	0.83 (0.55–1.25)	0.81 (0.28–2.32)	0.49 (0.17–1.38)	0.3 (0.11–0.81)^*∗*^
>=5	2.2 (1.06–4.56)	1.94 (0.91–4.14)	2.42 (1.29–4.54)	0.17 (0.02–1.34)	0.15 (0.02–1.03)	0.59 (0.07–4.65)
Vegetable intake/day						
0-1	1	1	1	1	1	1
2–4	1.67 (1.09–2.57)	1.07 (0.69–1.68)	1.28 (0.78–2.12)	0.64 (0.24–1.68)	0.3 (0.11–0.82)^*∗*^	0.29 (0.11–0.72)^*∗*^
>=5	2.07 (0.58–7.43)	1.67 (0.36–7.63)	0.78 (0.22–2.7)	1.13 (0.19–6.74)	2.46 (0.29–20.65)	4.86 (0.66–35.82)

^*∗*^ *P* < 0.05.

## Abbreviations

AOR: Adjusted odds ratio
BP: Blood pressure
BMI: Body mass index
CVD: Cardiovascular disorder
CI: Confidence interval
DASH: Dietary approaches to stop hypertension
DBP: Diastolic blood pressure
EA: Enumeration area
HTN: Hypertension
IHD: Ischemic heart disease
ISCO: International standard classification of occupation
JNC 7: Joint national committee 7 for hypertension
NCD: Noncommunicable disease
NCEP: ATP III: National Cholesterol Education Program: Adult Treatment Panel III
OR: Odds ratio
PSU: Primary selection unit
SA: South Africa
SAGE: Study on global AGEing and adult health
SBP: Systolic blood pressure
WC: Waist circumference
WHO: World Health Organization.
